# Effect of Occlusal Reduction on Post-operative Pain in Diabetic and Non-diabetic Patients With Symptomatic Apical Periodontitis in a Single Visit: An In Vivo Study

**DOI:** 10.7759/cureus.94754

**Published:** 2025-10-16

**Authors:** Aisha S Baig, Panna Mangat, Gauravi Bajpai, Bhaviya Chandel, Mampi Biswas, Viswesh S, Iman Baig

**Affiliations:** 1 Conservative Dentistry and Endodontics, Kalka Dental College, Meerut, IND; 2 Periodontology, King George's Medical University, Lucknow, IND

**Keywords:** diabetes mellitus endodontic treatment, occlusal reduction, postoperative pain, single-visit root canal treatment, symptomatic apical periodontitis

## Abstract

Background: Pain is a common and significant sensory and emotional experience that often drives patients to seek medical care. In dentistry, managing pain, especially during and after endodontic treatment, remains a critical challenge. Endodontics focuses on treating diseases of the dental pulp and periradicular tissues. Although root canal treatment (RCT) is often perceived as painful, studies indicate that patients who have undergone RCT frequently describe it as painless. Despite advancements, postoperative pain due to acute periapical inflammation is still a concern. Apical periodontitis, often resulting from pulp infection, can also be influenced by systemic conditions such as diabetes mellitus, which is associated with impaired healing and increased risk of periodontal disease. Occlusal reduction, a technique to minimise mechanical stimulation of sensitised nociceptors, may reduce postoperative pain by decreasing pressure on inflamed periapical tissues.

Objective: This study aimed to evaluate the effect of occlusal reduction on postoperative pain in diabetic and non-diabetic patients with symptomatic apical periodontitis undergoing single-visit endodontic treatment.

Hypothesis: Occlusal reduction reduces postoperative pain in both diabetic and non-diabetic patients following single-visit RCT.

Methods: This study included 90 patients with symptomatic apical periodontitis (30 diabetic and 60 non-diabetic). Sensibility of the tooth was checked using a cold test and an electric pulp tester. Diagnosis was confirmed through tenderness on percussion and radiovisiography. Glycaemic control in diabetic patients was assessed using HbA1c levels. Randomisation was performed in a 1:2 ratio (diabetic: non-diabetic). Patients recorded pain intensity on a visual analogue scale at 1, 6, 12, 24, 48, and 72 hours, and 1 week post-treatment. The PICO format guided the study design. Data were analysed statistically. RCT was performed with ProTaper Gold rotary files (Dentsply Maillefer, Switzerland), sodium hypochlorite or ethylenediaminetetraacetic acid irrigation, single cone obturation, occlusal reduction, and composite restoration after 72 hours. All patients were prescribed a combination analgesic containing aceclofenac 100 mg, paracetamol 325 mg, and serratiopeptidase 15 mg, to be taken only in case of moderate-to-severe pain.

Result: Data were analysed using SPSS version 23.0 (IBM Corp., Armonk, NY), with descriptive statistics including mean, standard deviation, frequency, and percentage. The Mann-Whitney U test was used for intergroup comparisons, and the Wilcoxon signed-rank test for intragroup comparisons. Normality was assessed using the Shapiro-Wilk test, revealing non-normal data distribution, necessitating non-parametric analysis. In the diabetic group, pain peaked at 6 hours (mean = 2.933) and reduced significantly by 48 hours (mean = 0.333), with no pain at 72 hours and 1 week. The non-diabetic group showed a similar trend, with peak pain at 6 hours (mean = 3.083) and no pain by 72 hours. Intergroup analysis showed a significant difference only at the 1-hour mark (p = 0.001), with non-diabetic patients experiencing higher pain.

Conclusion: Both diabetic and non-diabetic groups showed significant pain reduction over 72 hours, with the most substantial decrease within the first 48 hours. The pain patterns were comparable between groups except at 1 hour, where non-diabetics reported higher pain.

## Introduction

Pain is defined as “an unpleasant sensory and emotional experience associated with actual or potential tissue damage” and remains the primary reason patients seek endodontic treatment [1]. Endodontic therapy encompasses the management of preoperative, intraoperative, and postoperative symptoms, with postoperative pain being a frequent complication that may progress to chronicity and represents a major concern for patients [2].

Postoperative pain may arise from several etiological factors, including chemical irritation due to extrusion of irrigants or medicaments, mechanical causes such as over-instrumentation or extrusion of filling materials, and microbial injury to periapical tissues, resulting in acute inflammation [3].

Apical periodontitis is an inflammatory reaction at the root apex, usually following microbial infection of the pulp space. It is a host immune response of the periapical tissues to gram-negative bacterial invasion of the pulp, most often (about 90% of cases) secondary to pulpal necrosis from dental caries. Apical periodontitis is not merely a localised condition; its possible systemic implications have led to the emergence of “endodontic medicine,” which investigates links between periapical pathology and systemic diseases [4].

Diabetes mellitus (DM) is a chronic metabolic disorder with multiple pathophysiological consequences. There is strong recent evidence that diabetes and periodontitis share a bidirectional relationship, where poor glycaemic control exacerbates periodontal breakdown and periodontal inflammation can worsen glucose regulation [5,6].

One of the most important objectives in endodontic practice is to manage pain both during and after treatment. Different strategies have been suggested, including the use of preoperative analgesics and corticosteroids, administration of long-acting anaesthetics, and occlusal reduction [7]. Postoperative pain is primarily a result of acute periradicular inflammation and has been reported in 3%-58% of endodontically treated cases. Its causative factors include periapical inflammation, disruption of endodontic microbiota, and iatrogenic mechanical or chemical irritation [8].

Occlusal reduction has traditionally been advocated to minimise postoperative pain in root canal-treated teeth. The rationale is that eliminating occlusal contacts reduces mechanical stimulation of sensitised periodontal ligament nociceptors, thereby decreasing pressure on inflamed periapical tissues and alleviating mechanical allodynia [9]. The potential benefits of occlusal reduction have been evaluated in pulpal and periapical pathologies, with studies comparing outcomes between teeth receiving complete occlusal reduction (where all biting contacts are removed to relieve pressure on inflamed tissues) and those undergoing occlusal adjustment (where only minor selective corrections are made to maintain normal functional contacts). The goal in both approaches is to lower occlusal load and reduce postoperative discomfort.

## Materials and methods

This study was designed as a prospective, comparative, in vivo study at the Department of Conservative Dentistry and Endodontics, Kalka Dental College and Hospital, Meerut, Uttar Pradesh, India, from June 2023 to December 2023. The study protocol adhered to the ethical standards outlined in the 1964 Declaration of Helsinki as revised in 2008. Ethical approval was obtained from the Institutional Ethical Committee of the Kalka Dental College, Meerut (approval number KDC/LTR/2023/0109). Before enrolment, all patients were provided with detailed information about the study’s purpose, procedures, potential risks, and benefits. Written informed consent was obtained using standardised consent forms, ensuring voluntary participation and the right to withdraw at any time without any consequences.

The required sample size was determined a priori using the G*Power software (version 3.1.9.2; Heinrich Heine University, Düsseldorf, Germany). Based on the 90% power of the study, 5% type I error, and effect size of 0.60 (effect size for the significant difference between the groups), the minimum sample size came out to be 90 patients, i.e., 30 in the study group and 60 in the control group [10].

Inclusion criteria

Participants included individuals diagnosed with symptomatic apical periodontitis in multirooted teeth (premolars or molars), aged between 18 and 65 years. The diabetic group consisted of patients with a minimum three-year history of type 2 DM and good glycemic control (HbA1c < 6.5%).

Exclusion criteria

Patients with systemic complications, severe pain or apical abscess, multiple teeth requiring treatment, teeth with severe periodontal disease or calcified canals, pregnant women, and those unwilling to consent or follow up were excluded.

Discontinuation criteria

Patients who were lost to follow-up or experienced procedural errors, such as instrument separation within the canal, ledge formation, or perforation during instrumentation, were excluded from the final analysis.

Study design

**Figure 1:**
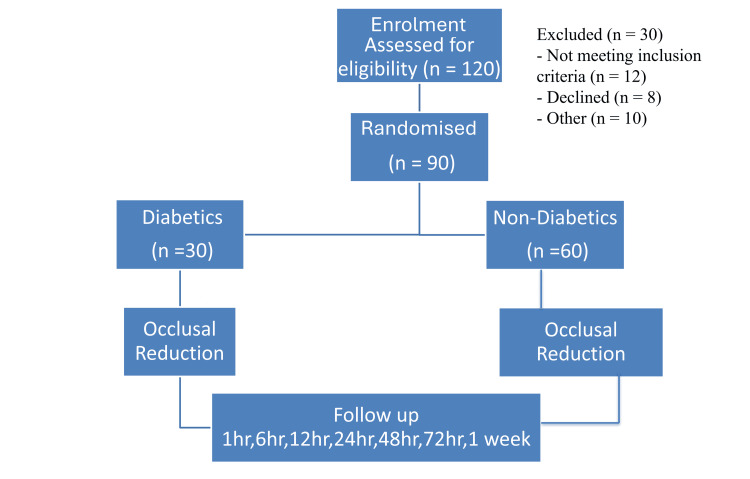
Study flowchart

A total of 90 individuals diagnosed with symptomatic apical periodontitis participated in the study, comprising 30 diabetic and 60 non-diabetic patients. Participants reported primary complaints of pain while biting and chewing in the posterior teeth. Sensibility was checked using a cold test and an electric pulp tester, while tenderness on percussion was assessed. Radiovisiography was conducted to confirm the diagnosis of symptomatic apical periodontitis, necessitating endodontic treatment. Before initiating treatment, patients were provided with a visual analogue scale (VAS) to evaluate their baseline pain levels. They received a comprehensive explanation of the study’s purpose, methodology, possible risks, and overall structure. Diabetic participants (with a minimum three-year history of type 2 diabetes) underwent HbA1c testing to evaluate their glycaemic control status. Block randomisation was employed, ensuring that for each diabetic participant, two non-diabetic participants were selected. The 1:2 randomisation ratio (30 diabetic and 60 non-diabetic patients) was determined using G*Power software version 3.1.9.2, accounting for the lower clinical prevalence of diabetic cases with symptomatic apical periodontitis and to maintain 90% study power at a 5% significance level. Each patient received a VAS form to document their pain levels at specific intervals: 1 hour, 6 hours, 12 hours, 24 hours, 48 hours, 72 hours, and 1 week post-endodontic treatment. Periodic reminders were sent via telephone to ensure participants completed their pain assessments at the designated times (Figure 1).

Clinical procedure

Patients presented with complaints of biting or chewing pain. Diagnosis of symptomatic apical periodontitis was confirmed through sensibility tests (cold test and electric pulp tester), percussion, and radiovisiography. All patients underwent standardised single-visit root canal treatment (RCT), as this approach minimises interappointment microbial contamination, enhances patient compliance, and allows more accurate assessment of postoperative pain within a controlled time frame. A total of 90 teeth with symptomatic apical periodontitis in premolars and molars were selected for this study. The procedure was explained to the patients, and consent was taken from them. Administration of regional anaesthesia followed by rubber dam isolation was executed. Caries excavation, if present, and access cavity preparation were performed. Working length determination was carried out with 10 no. K-file using an electronic apex locator, and confirmation was conducted using a radiograph. Shaping was carried out by using ProTaper Gold rotary files (Dentsply Maillefer, Switzerland). For maxillary first and second premolars, shaping was performed till the F2 file. For maxillary first and second molars, mesiobuccal and distobuccal canals were prepared till F1 and palatal till F2. For mandibular first and second molars, mesiobuccal and mesiolingual canals were prepared till F1 and distal till F2 (if two distal canals were present, they were prepared till F1 each). Cleaning or irrigation was performed using 3% sodium hypochlorite (10 mL per canal), and 17% ethylenediaminetetraacetic acid was used alternatingly after instrumentation with each file system. After completion of chemo-mechanical preparation, normal saline was used as a final irrigant. Canals were dried using paper points. Obturation was performed using respective gutta-percha cones with calcium hydroxide-based sealer (CalApex, Prevest Denpro, Jammu) using the single cone obturation technique. Temporary restoration was placed. Occlusal reduction was performed in all patients. Post-endodontic restoration was performed with composite filling material after 72 hours. All patients were prescribed Zerodol-SP (aceclofenac 100 mg, paracetamol 325 mg, and serratiopeptidase 15 mg) and instructed to take only if they experienced moderate-to-severe pain. The evaluation of post-obturation pain was performed with VAS [11].

Statistical analysis

Data were recorded in Microsoft Excel 2007 (Microsoft Corp., Redmond, WA, US) and analyzed using SPSS version 23 (IBM Corp., Armonk, NY). Descriptive statistics (mean, standard deviation (SD), frequency, and percentage) were used to summarise findings. The Mann-Whitney U test was applied to compare post-operative pain scores between the two groups. A p-value of ≤0.05 was considered statistically significant. All analyses were conducted on the final dataset after excluding participants lost to follow-up or those with procedural errors. No missing data were included in the statistical evaluation, and complete case analysis was performed to maintain data integrity.

## Results

The descriptive statistics included mean, SD, frequency, and percentage. The level of significance for the present study was fixed at 5%. The intergroup comparison was carried out using the Mann-Whitney U test, and the intragroup comparison between time intervals was performed using the Wilcoxon signed rank test.

The baseline characteristics of the study groups were comparable. The mean age of diabetic males was 49.38 ± 6.67 years, while diabetic females had a mean age of 47.93 ± 5.68 years; the difference was not statistically significant (t = 0.64, p = 0.526). Sex distribution showed that in the diabetic group, 16 (53.3%) were males and 14 (46.7%) were females, whereas in the non-diabetic group, 28 (46.7%) were males and 32 (53.3%) were females. Chi-square analysis confirmed no significant difference in sex distribution between the groups (χ² = 0.14, p = 0.709) (Table 1 ).

These findings confirmed that there was no demographic bias in terms of age or sex, ensuring comparability between groups.

**Table 1:** Demographic characteristics of the study population

Parameter	Male	Female	Test statistic	p-value
Age (years)	49.38 ± 6.67	47.93 ± 5.68	0.64	0.526
Sex distribution (diabetic)	16 (53.3%)	14 (46.7%)	0.14	0.709
Sex distribution (non-diabetic)	28 (46.7%)	32 (53.3%)	0.14	0.709

The analysis of pain levels in the diabetic group at various time intervals revealed significant changes in pain intensity (Table 2). At the 1-hour mark, the mean pain score was 1.667 (SD = 0.422), indicating a mild level of discomfort. Pain levels increased significantly at 6 hours, with a mean score of 2.933 (SD = 0.740), but decreased at 12 hours to a mean of 1.733 (SD = 0.980). By 24 hours, the mean pain score dropped further to 1.216 (SD = 1.747). At 48 hours, the mean score decreased significantly to 0.333 (SD = 0.884), and no pain was reported at the 72-hour and 1-week mark, with a mean score of 0.000 (SD = 0.000).

The analysis of pain in the non-diabetic group at various time intervals revealed notable changes in pain intensity (Table 3). At the 1-hour mark, the mean pain score was 2.150 (SD = 1.273), reflecting mild-to-moderate discomfort. Pain levels rose to a mean score of 3.083 (SD = 1.730) at 6 hours, before dropping at 12 hours to 1.816 (SD = 1.171). By 24 hours, the mean pain score decreased further to 1.291 (SD = 1.866), and at 48 hours, the mean score significantly dropped to 0.400 (SD = 0.924). Like the diabetic group, no pain was reported at the 72-hour mark and 1 week, with a mean score of 0.000 (SD = 0.000).

**Table 2:** Pain in the diabetic group at different time intervals

	N	Mean	Standard deviation	Standard error	Minimum	Maximum
1 hour	30	1.667	0.422	0.259	0	6
6 hours	30	2.933	0.74	0.317	1	6
12 hours	30	1.733	0.98	0.178	1	4
24 hours	30	1.216	1.747	0.225	0	6
48 hours	30	0.333	0.884	0.161	0	3
72 hours	30	0	0	0	0	0
1 week	30	0	0	0	0	0

**Table 3:** Pain in the non-diabetic group at different time intervals

	N	Mean	Standard deviation	Standard error	Minimum	Maximum
1 hour	60	2.15	1.273	0.164	0	6
6 hours	60	3.083	1.73	0.223	1	6
12 hours	60	1.816	1.171	0.151	0	4
24 hours	60	1.291	1.866	0.17	0	6
48 hours	60	0.4	0.924	0.119	0	3
72 hours	60	0	0	0	0	0
1 week	60	0	0	0	0	0

Mann-Whitney U test for the intergroup comparison

The intergroup comparison of pain between the diabetic and non-diabetic groups at different time intervals showed varying results (Table 4).

**Table 4:** Intergroup comparison of pain in the diabetic and non-diabetic groups at different time intervals

	Group	N	Mean	Standard deviation	Standard error mean	p-value	Significance
1 hour	Diabetic	30	1.667	0.422	0.259	0.001	Significant
	Non-diabetic	60	2.15	1.273	0.164		
6 hours	Diabetic	30	2.933	0.74	0.317	0.321	Non-significant
	Non-diabetic	60	3.083	1.73	0.223		
12 hours	Diabetic	30	1.733	0.98	0.178	0.658	Non-significant
	Non-diabetic	60	1.816	1.171	0.151		
24 hours	Diabetic	30	1.216	1.747	0.225	0.63	Non-significant
	Non-diabetic	60	1.291	1.866	0.17		
48 hours	Diabetic	30	0.333	0.884	0.161	0.744	Non-significant
	Non-diabetic	60	0.4	0.924	0.119		
72 hours	Diabetic	30	0	0	0	1	Non-significant
	Non-diabetic	60	0	0	0		
1 week	Diabetic	30	0	0	0	1	Non-significant
	Non-diabetic	60	0	0	0		

At the 1-hour mark, the mean pain score in the diabetic group was 1.666 (SD = 0.422), while the non-diabetic group had a higher mean of 2.150 (SD = 0.273). This difference was statistically significant, with a p-value of 0.001. However, at the subsequent time intervals, no significant differences were observed between the two groups. At 6 hours, the mean pain scores were 2.933 (SD = 0.740) for diabetics and 3.083 (SD = 0.730) for non-diabetics, with a p-value of 0.321, indicating no significant difference. Similarly, at 12 hours, the diabetic group had a mean of 1.733 (SD = 0.980) and the non-diabetic group had 1.816 (SD = 1.171), with a p-value of 0.658, also non-significant.

**Figure 2:**
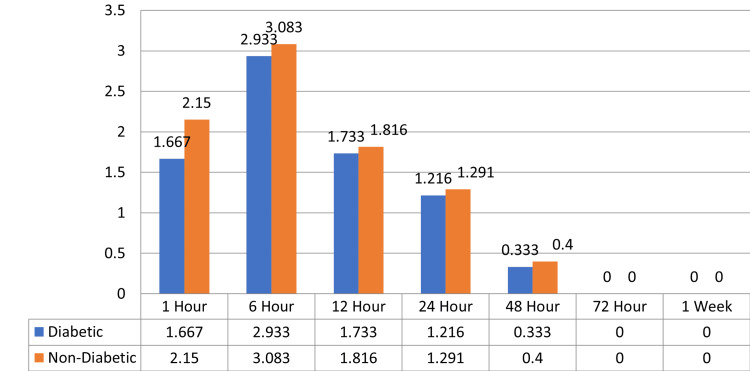
Bar graph representing mean pain scores at different time intervals in diabetic and non-diabetic patients.

At 24 hours, the diabetic group had a mean pain score of 1.216 (SD = 1.747), and the non-diabetic group had 1.291 (SD = 1.866), with a p-value of 0.630, indicating no significant difference. At 48 hours, the mean pain scores for diabetics and non-diabetics were 0.333 and 0.400, respectively, with a p-value of 0.744, also non-significant. Similarly, at 72 hours and 1 week, the pain scores for the two groups were 0.000 for diabetics and non-diabetics, with a p-value of 1.000, indicating no significant difference. In conclusion, while there was a significant difference between the diabetic and non-diabetic groups in terms of pain at 1 hour, no significant differences were found at the other time intervals (6, 12, 24, 48, and 72 hours and 1 week) (Figure 2).

## Discussion

This in vivo study evaluated the effect of occlusal reduction on postoperative pain in diabetic and non-diabetic patients undergoing single-visit RCT for symptomatic apical periodontitis. By comparing pain levels between the two groups, the study sought to determine whether occlusal trauma contributes significantly to post-endodontic pain and whether systemic conditions such as diabetes influence pain response and healing. The findings revealed a similar trend of pain reduction in both groups, with a statistically significant difference only at the one-hour interval, indicating that occlusal reduction effectively minimises early postoperative discomfort irrespective of diabetic status.

Comparable postoperative pain trajectories between diabetic and non-diabetic patients have also been reported in earlier randomized trials and observational studies [10,12]. Controlled diabetic patients tend to experience pain outcomes similar to non-diabetic individuals following RCT, whereas poorly controlled diabetes has been associated with delayed periapical healing and a higher risk of persistent lesions [4,12]. These results reinforce the critical role of good glycaemic control in achieving favorable endodontic outcomes among diabetic patients.

The present results align with the concept of endodontic medicine, which emphasizes the bidirectional relationship between systemic health and periapical disease [4]. Diabetes has been shown to modulate host immune responses, impair neutrophil chemotaxis, and reduce macrophage activity, collectively slowing the resolution of periapical inflammation [13]. Persistent hyperglycaemia also alters microvascular circulation, thereby compromising nutrient and oxygen delivery to periapical tissues.

The lower immediate postoperative pain observed in the diabetic group may be explained by diabetes-related alterations in nociceptive signaling. Peripheral neuropathy, a frequent complication of chronic diabetes, causes degeneration of small-diameter nerve fibers and diminished pain perception (hypoalgesia) [9]. Hyperglycemia-induced advanced glycation end products interfere with axonal transport and neurotransmitter release, attenuating acute pain transmission [9,12]. Additionally, the inflammatory response is often blunted in diabetes because of reduced cytokine recruitment, which could result in less pronounced pain following endodontic instrumentation [12].

With respect to occlusal management, our findings correspond to earlier research demonstrating that occlusal reduction decreases postoperative discomfort in symptomatic cases [7,14]. Rosenberg et al. first reported that complete removal of occlusal contacts significantly reduced post-treatment pain in teeth with pre-operative sensitivity [7]. Fathy et al. and Nguyen-Nhon et al. confirmed these benefits through randomized trials and meta-analyses [14,15]. However, Vianna et al. and Manigandan et al. found no statistically significant differences in pain levels after occlusal adjustment, suggesting that the benefit may be short-term and case-specific [16,17]. Variations in case selection (vital vs. necrotic pulps), instrumentation techniques, and patient factors such as systemic health could explain discrepancies among studies.

Clinically, these findings suggest that clinicians should consider implementing occlusal reduction, especially in diabetic patients undergoing single-visit endodontic treatments for symptomatic apical periodontitis, to enhance postoperative comfort and potentially improve healing outcomes. Additionally, a comprehensive assessment of a patient’s systemic health, particularly diabetic status, is crucial in predicting postoperative pain and customizing endodontic interventions accordingly. Overall, incorporating occlusal reduction as a routine step in the management of symptomatic cases, alongside strict glycemic monitoring, can significantly improve patient comfort, reduce postoperative complications, and promote faster recovery.

However, this study is not without limitations. Although only diabetic patients with controlled HbA1c levels and no self-reported systemic complications were included, specific screening for diabetic neuropathy was not conducted. As peripheral neuropathy can alter pain perception and lead to hypoalgesia, its presence may have introduced bias in postoperative pain assessment among diabetic participants. Additionally, the study was conducted at a single center with a relatively short follow-up period, focusing primarily on immediate postoperative pain rather than long-term healing outcomes. These factors may limit the generalizability of the findings. Future research should include multicenter trials with larger and more diverse populations, incorporate objective neurological screening to rule out diabetic neuropathy, and extend observation periods to evaluate the long-term effects of occlusal reduction on periapical healing and patient comfort.

## Conclusions

In conclusion, while occlusal reduction remains an essential step in the management of symptomatic apical periodontitis, its clinical application should be modified according to patient-specific parameters. Both diabetic and non-diabetic patients benefited from occlusal reduction, experiencing a comparable decline in postoperative pain. For diabetic patients, maintaining good glycaemic control is crucial to enhance healing and reduce postoperative complications. Clinically, these findings support the inclusion of occlusal reduction as a routine adjunct in endodontic therapy to minimise postoperative discomfort and promote favourable outcomes across different systemic health profiles. These insights are instrumental in guiding future clinical practice and optimising patient-centred dental care.
